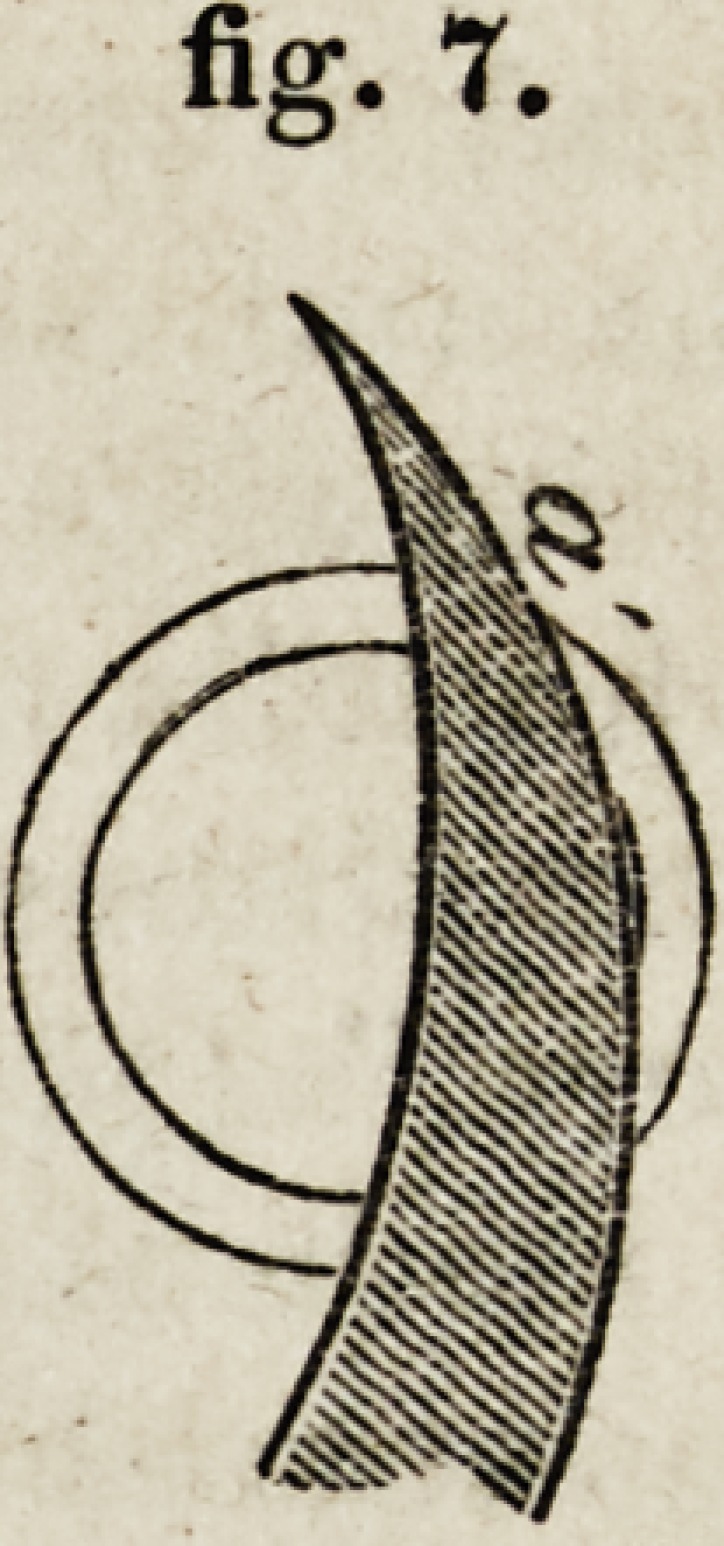# Cataract, and Its Treatment; Comprising an Easy Mode of Dividing the Cornea for Its Extraction, and Appropriate Means for Removing the Different Forms of That Affection

**Published:** 1844-04

**Authors:** 


*Cataract, and its Treatment, comprising an easy mode of dividing the Cornea for its extraction, and appropriate means for removing the different forms of that Affection.* By John Scott, Senior Surgeon to the Royal London Ophthalmic Hospital, &c.—*London,* 1843. 8vo, pp. 72.

Almost the only original part of this pamphlet is contained in the two or three pages preceding and following the woodcuts by which it is accompanied. The rest, with very little exception, is made up of such remarks on the symptoms, varieties, diagnosis, causes, prognosis, and treatment of cataract, as are to be found in all the common treatises on disorders of the eye; and our wonder is, that a gentleman of Mr. Scott's standing in the profession should stoop thus "to twist the same rope again." As, however, some of the subjects discussed in Mr. Scott's production are of the greatest importance in a practical point of view, we shall give to its contents a fuller consideration than either its size or its intrinsic merits would otherwise demand.

The following are a few particulars which deserve notice in the early part of the work.

Mr. Scott bears testimony (p. 2) to the existence of what has been called *black cataract.* "I have removed," says he, "a cataract nearly as dark as pitch, and it was of very firm consistence."

Speaking of soft cataract, (p. 10,) he tells us, that it "occurs at an earlier period of life than hard cataract," and that "the margin of the lens is usually more opaque, so that vision is more obscured even in a dilated state of the pupil." The latter statement affords a specimen of a style of writing, ambiguous from carelessness, which occurs pretty frequently in Mr. Scott's pamphlet. Does he mean, that in soft cataract vision is more obscure, even with a dilated pupil, than it is in hard cataract; or that vision is more obscure, even in a dilated state of the pupil, than in a contracted state? As to soft cataract occurring at an earlier period of life than hard cataract, it might have been well to have stated the cause, viz. the difference in the natural consistence of the lens in young and in old people.

At page 18, Mr. Scott says, "In the early stage of cataract, I am aware that the lens still retains its natural consistence, and that the operation by solution will then effect its removal," a remark which might lead the reader to suppose that the duration of a cataract, independently of the advances of age, had a tendency to harden the lens, and that the operation of division was an advisable one, even in old people, if the opacity had only recently made its appearance, neither of which suppositions would be agreeable to fact.

Mr. Scott does not think it desirable to operate on both eyes at the same time, for the following very satisfactory reasons, which we have not seen insisted on so closely and forcibly by any other author:

"Should there," says he, "be any unhealthy state of system causing inflammation to succeed the operation, it will equally influence both eyes, and vision may be irrecoverably lost; or if, from accidental local circumstances, inflammation be set up in one eye, the second may very probably be sympathetically affected; whereas, if one only be operated on, and any unfavorable result should occur, we may hope to operate on the other under more favorable circumstances, and with good prospect of success." (p. 19.)

At page 25, Mr. Scott tells us, that the pupillary margin of the iris is more liable to be divided by the knife in the dilated than in the contracted state; an opinion contrary, we believe, to what is generally held. But granting Mr. Scott's statement to be correct, it seems to us to militate against the advice previously given, (p. 22,) that, in cases of smallness of the anterior chamber, the pupil should be fully dilated, so as *to obtain room to pass the knife without wounding the iris.*

Mr. Scott does not appear sufficiently guarded in two statements which he makes regarding the escape of the aqueous humour, in the operation of division through the cornea; for at page 22 his words are, "This operation by solution, however, being also generally attended with the escape of the aqueous humour," whereas at page 35 he says, "The introduction of a needle into the anterior chamber can always be effected without the slightest difficulty, and it can generally be retained there for a sufficient length of time to break up the texture of the lens without the escape of the aqueous humour." To this criticism, it might perhaps be objected that, at page 22, our author refers only to cases of small anterior chamber, but at page 35, to eyes of normal conformation. We doubt, however, if even this explanation can acquit him of contradiction; for it is not upon the amplitude of the anterior chamber, but on the form of the needle, and the method of handling it, that the confinement of the aqueous humour depends.

The recumbent position is recommended (p. 26) for the operation of extraction, as one which not only obviates most effectually any unsteadiness on the part of the patient, but allows the surgeon to rest his hand in an easy and convenient manner, and thus enables him to perform the section with precision. Mr. Scott subjoins another reason, of which we are not so sure. "It presents," says he, "the escape of the vitreous humour to any deleterious extent, for it cannot gravitate out of the eye in this position." We have certainly seen the vitreous humour fly from the eye as frequently when the patient was lying as sitting, and think it pretty certain that gravitation has nothing to do in the matter.

The following judicious advice refers to the third period of the operation or the exit of the lens:

"In this part of the operation it is necessary to take care that the pressure is confined to the anterior part of the eye, which is to be compressed just behind the margin of the lens, so as to dislodge it from the capsule, and to tilt it forward through the pupil; if the whole globe of the eye be pressed backwards into the orbit, the escape of the vitreous humour will be endangered, instead of the protrusion of the lens taking place." (p. 30.)

It sometimes happens, that in making the section of the cornea, a small portion of the iris is excised. If this portion does not include the pupillary edge, a circular aperture will be formed in the iris, and the pupil, Mr. Scott informs us, will not dilate readily when the lens is pressed against it. He therefore divides, with Maunoir's scissors, the fibres intervening between such an aperture and the natural pupil, thus allowing the cataract to be extracted. (p. 33.) We do not recollect to have met with this rule in any other author.

For enlarging the section of the cornea, when it happens to be made too small, we agree with Mr. Scott, that Maunoir's scissors is not an appropriate instrument. David's doubly-curved scissors have the advantage of cutting close and parallel to the edge of the cornea, and are greatly superior to the knife recommended (p. 33) by Mr. Scott, for enlarging the incision.

The idea (p. 35) of returning the vitreous humour into the eye, with the curette, is natural enough; but the thing is impracticable.

Mr. Scott recommends a narrow convex-edged knife for making the section of the cornea, very different in shape, therefore, from the knives of Wenzel and Beer, but not very unlike those employed by the Pelliers, the sons of him whose speculum was so long in vogue. The forms of the Pelliers' knives are delineated in fig. 1 of plate viii, and fig. 6 of plate xxiii, of Pellier de Quengsy's 'Cours d'opérations sur la chirurgie des Yeux,' and the reader may compare them at his leisure with Mr. Scott's knife, of which the following figure (fig. 1) shows the shape and dimensions.

fig. 1.

Mr. Scott is not the first who has felt that there are difficulties and dangers in dividing circularly with a knife a membrane, inclosing a fluid, which is not to be evacuated till the section is carried to the extent of a semicircle, and within which fluid is suspended another and a moveable membrane, which is neither to be allowed to be displaced, nor is in any way to be injured, in the execution of this section. Such titles as 'An Inquiry into the Causes which have most commonly prevented success,' or 'A Practical Inquiry into the Causes of the Frequent Failure,' prefixed to writings by Ware and Adams, are sufficient to show that extraction, even in the hands of oculists of great experience, is far from being an operation, the results of which can be calculated on with certainty. Thousands of eyes have, doubtless, been destroyed in this operation since the days of Ware and Adams; aye, even in spite of "The Certainty and Safety with which the Operation for the Extraction of a Cataract may be performed, by G. J. Guthrie, F.R.S." The difficulties and dangers attendant on extraction are indissolubly connected with the nature of the parts concerned, and the extent and object of the operation; they are often aggravated by the unhealthy condition of other structures in the eye, besides those immediately implicated; and, though there is a wide difference between the workmanship of a steady and dexterous operator, and that of a bungler, too conceited to be taught, and too little honest to refrain from what he has not brains to comprehend, we are convinced that the dangers and difficulties of extraction never can be set aside, and can, only in a very moderate degree indeed, be obviated by giving to the instrument with which the cornea is to be divided, the very best form which ingenuity and experience are able to suggest.

Mr. Scott is rather of a different opinion. He tells us, in his preface, that he has "long considered that the chief difficulty and the chief danger attending the extraction of cataract has arisen from the force which is necessary to transfix the cornea with the instruments commonly employed for that purpose; and that spasm of the recti muscles, induced by that force compressing the iris between the hard lens and the side of the knife, and occasioning inflammation of that membrane, has been the most frequent cause of an unfavorable result of the operation." It therefore occurred to him, "that if these inconveniences could be obviated," in other words, if a knife could be invented, which should require less force to transfix the cornea than those of Wenzel and Beer, "the facility of performing the operation would be greatly increased, and its success proportionately augmented." With this view, then, Mr. Scott has constructed a knife, and having tested it in a great number of cases, he feels anxious to afford to others an opportunity of employing it.

"The cornea-knives usually employed," says Mr. Scott, "not only increase in thickness and in width from point to heel, to fill up the aperture they make in the cornea, as they traverse the anterior chamber, and thus prevent the escape of the aqueous humour, but their width is also equal to the radius of the cornea, so as to make a section of that size in the membrane; and this is done by thrusting this wedge-shaped instrument through the cornea, the cutting edge of the knife effecting its division by means of the force with which the back of the instrument is pressed against the opposite margin of the wound. This forcible thrusting of a wedge shaped instrument of such dimensions through the anterior chamber appears to me to be productive of many of the difficulties as well as the dangers that attend the operation. Thus the force employed tends to turn the eye inwards to the nasal canthus of the orbit, whereby the inner side of the cornea is obscured from the view of the operator, he is unable to puncture it close to its sclerotic margin, and consequently the section is too small for the escape of the cataract.

"If this inversion of the eye is prevented by pressure on the nasal side of the globe, the aqueous humour is liable to escape before the knife has traversed the anterior chamber far enough to prevent the iris from being wounded in completing the section; and even if the knife be so far advanced that the iris cannot escape beneath its edge, the pressure necessarily exerted on the globe often induces such violent spasm of the muscles as to endanger the escape of the vitreous humour, and to subject the iris and the internal tunics to so much pressure as to lay the foundation of serious inflammation." (p. 34.)

Mr. Scott appears to have Beer's knife chiefly in view when he states that the common cornea-knives increase in thickness and in width from the point to the handle. This is a property which has generally been regarded as a valuable one, the knife being thereby enabled to fill up the aperture in the cornea as it advances, and thus to prevent the premature escape of the aqueous humour; and accordingly Mr. Scott studies to preserve this property in the knife which he himself has adopted.

The accusation brought against the common knives, that their breadth is equal to the radius of the cornea, is just; but the reason for this breadth is not "to make a section of that size in the membrane," as Mr. Scott expresses it, but to ensure a semicircular section of the cornea, by the mere progression of the instrument, without pressing or drawing it in the direction of the incision, and still more without sawing with it backwards and forwards, as must be done if the knife is narrow. A section of the size of the radius of the cornea, as Mr. Scott has it, does not express this fact, and probably does not express the meaning Mr. Scott had in view.

Mr. Scott seems to think it an objection to Beer's knife, that the cutting edge effects the section by its back resting against the undivided portion of the cornea, at the two opposite extremities of the incision. Is it not plain that, unless the back of the knife is in contact with that part of the cornea which remains entire, it will be almost impossible for the aqueous humour to be retained? We have no experience of narrow knives, such as Mr. Scott's, but if they execute the section on any other principle than the very one here objected to, we conceive they must expose the eye to great danger, from the premature loss of the aqueous humour.

The edges of Beer's knife being straight, and meeting at a small angle, it is mathematically demonstrable that it will suffer less resistance than a curved-edged instrument, be the curves what they may. Mr. Scott's expressions, then, of "thrusting this wedge-shaped instrument," and "this forcible thrusting," go for nothing. This cornea-sabre will require the employment of more force to make it transfix the cornea, than any straight-edged instrument, unless it be reduced to such a degree of narrowness in the blade, as will incapacitate it for the end of dividing the cornea, semicircularly, at the distance of 1-20th inch from its circumference, by simple progression; and reduce it from a knife to a saw, which is to haggle through the cornea, by being drawn backwards and forwards.

We happen to have known in our day and conversed with some of the most eminent oculists in this and other countries, who were in the habit of using Beer's knife, and we never heard them speak of the necessity of any "forcible thrusting" in the use of that instrument. On the contrary, everything like violence was always declared to be unnecessary and improper. We are therefore led to regard the objections raised by Mr. Scott as too much in the spirit of a special pleading. We can conceive his objections applicable only to those ill-contrived modifications of Beer's knife, by which its edges are made to meet at a greater angle than 15°. Then, indeed, it is plain it will both traverse the cornea less easily, and, from the slowness with which it must move, expose the eye to a premature loss of the aqueous humour.

Mr. Scott seems of opinion that to apply the point of a finger against the nasal side of the eyeball, so as to prevent it from rolling inwards, should be abandoned, as he thinks such pressure causes the aqueous humour to escape, before the knife has traversed the anterior chamber. We are completely of the opposite opinion. We believe a frequent cause of the accident in question is the patient's being allowed to turn his eye suddenly inwards, at the moment of entering the knife. The plan of operating without making any pressure on the eyeball has often been tried,* but for the reason now mentioned, as well as the difficulty of effecting a sufficient incision of the cornea if the eye is allowed to roll inwards, it is now almost universally and very wisely abandoned.

* Ware's Observations on the Cataract, &c., p. 273; London, 1812.

Another accusation brought forward by our author is, that "the pressure necessarily exerted on the globe often induces such violent spasm of the muscles as to endanger the escape of the vitreous humour," to which we have only to reply, that any such violent pressure is perfectly unnecessary for any purpose, and especially for the purpose specified, namely, steadying the eyeball and preventing it from turning into the nasal canthus.

We must repeat our impression, that a great portion of the objections raised by Mr. Scott against such instruments as are in common use at the present day for opening the cornea, are much exaggerated, and, in a great measure, groundless.

In the following passage Mr. Scott goes on to explain what he conceives to be the principle on which his cataract-knife is constructed:

"The introduction of a needle into the anterior chamber can always be effected without the slightest difficulty, and it can generally be retained there for a sufficient length of time to break up the texture of the lens without the escape of the aqueous humour, notwithstanding the repeated movements of it that are necessary for performing this operation. From reflecting on this circumstance, it occurred to me, that if a knife could be constructed that might be introduced into the eye with as little force as is necessary for the introduction of the needle, and could be formed of such a shape as would complete the section of the cornea without danger of wounding the iris, the difficulties and the danger attending the operation would be most materially lessened. Let it be remembered, that in the usual way of operating, the knife cuts its way *into* the cornea, which requires considerable force; whereas, upon the plan I propose, it is introduced into the anterior chamber without any further division of the cornea than is necessary for the purpose of its introduction, the section of the membrane not being commenced until both sides of the cornea have been punctured; and the knife is of such a shape and is then so situated that there is little danger of the iris falling forward before its edge." (p. 35.)

The introduction of a straight needle into the anterior chamber can always be effected without the slightest difficulty, but it is not so with a curved needle. A round-necked needle can generally be retained in the anterior chamber for a sufficient length of time to break up the lens without the escape of the aqueous humour; but it is quite otherwise if the neck of the needle is two-edged. To draw the conclusion, then, from such data, that a curved two-edged knife could be managed like a straight round-necked needle, is a bad specimen of Mr. Scott's logic. He tells us, further, that his plan is to have a knife which shall transfix the cornea before the proper section of the membrane is commenced; but it is sufficient just to glance at the actual breadth of his knife, gradually increasing from the point to the handle, (see fig. 1, above,) and at the figure in his fourth plate, to see that this proposed plan is contradicted in practice. It is quite undeniable that Mr. Scott's knife "is introduced into the anterior chamber without any further division of the cornea than is necessary for the purpose of its introduction;" but it is just as plain that, in transfixing the cornea, it must, from its breadth, divide a considerable portion of that membrane; that it cuts its way *into* the cornea; and that, from its curved form, it must require both more force and more sleight of hand than any straight-edged knife of equal or even of considerably greater breadth.

"The objects I propose to attain," says Mr. Scott, "in the construction of the knife are—

"1st. That it shall be of sufficient length to traverse completely the anterior chamber, and divide the nasal margin of the cornea.

"2d. That it shall increase in width and in thickness from point to heel enough only to prevent the escape of the aqueous humour in its transit across the anterior chamber, but that its width shall have no reference to the dimensions of the section that is to be made, as that circumstance, I conceive, has occasioned all the difficulty of its introduction, and the chief danger of the operation.

"3d. That it shall be of such a shape and figure, that when introduced in the middle of the temporal margin of the cornea and carried across the anterior chamber it shall readily puncture the nasal side of that membrane, and when placed in this situation the cutting edge shall be so far beyond the pupillary margin of the iris, and opposed to so large a portion of its anterior surface as will prevent its escape beneath the edge of the knife to endanger its division in making the section of the cornea.

"4th. That when the section of the cornea is thus about to be made, the edge of the knife shall be opposed only to the margin of the section on either side, and not to any extensive portion of its internal surface, whereby its division would be attended with difficulty, as is the case in using Beer's knife.

"In order to attain these objects, the knife must describe a portion of a circle of larger diameter than that of the cornea." (p. 56.)

"The back of the knife describes a sixth part of the circumference of a circle, the radius of which is ten lines. The cord of the arc formed by the back of the knife is, of course, also ten lines in length, being equal to the radius of that circle; it is therefore greater by four lines than the diameter of the cornea, and the blade is consequently quite long enough to complete the section of that membrane without difficulty. The knife is two lines in width at the heel, whence it gradually tapers to the point; it also increases uniformly in thickness, as well as in width, from point to heel, so as to occupy completely the aperture it makes in the cornea, for the purpose of preventing the escape of the aqueous humour." (p. 37.)

"In making the upper section of the cornea with this knife, it is to be held in the usual manner, between the thumb and two fore-fingers, the two other fingers resting on the patient's cheek, and the handle of the knife slightly inclined towards the side of the face, while the point punctures the cornea on its temporal margin; the handle of the knife is then to be brought upwards with a sweep as the blade traverses the anterior chamber; and when it has punctured the nasal side of the cornea, the angle will be nearly at a right angle with the temple. The knife is then to be carried completely across the anterior chamber: in doing this great care must be taken to press firmly downwards with the back of the instrument, so that the wound may not be unnecessarily enlarged by its cutting edge. This being accomplished, the point of the knife will have reached the nasal canthus of the orbit, and its cutting edge will be so far beyond the pupillary margin of the iris that it cannot be readily divided in completing the section of the cornea. The point of the knife is then to be carried upwards, the handle being slightly inclined in the opposite direction. The section of the cornea on its nasal side will now be complete, a small portion of the upper and outer part only remaining to be divided; and this is readily done in the withdrawing of the instrument." (p. 43.)

Such is Mr. Scott's mode of making the section of the cornea, which, in order that it may be exactly understood, it is necessary to analyse.

Suppose it is the right eye which is to be operated on, the patient lies on his back, the operator sits or stands behind him, holds the knife in his right hand, supports the upper eyelid with the fore and middle fingers of the left hand, takes great care not to permit the inversion of the eye towards the nose by pressure on the nasal side of the globe, and proceeds in the following manner to make the upper section.

First, the point of the knife is to be directed upwards and inwards, and the handle towards the patient's face. This position serves for making the puncturation. Secondly, the handle is to be brought up with a sweep, and the blade, by this manœuvre, is to traverse the anterior chamber. Thirdly, the counterpuncturation is to be effected, and the handle made to assume a position nearly at a right angle with the temple. Fourthly, the blade of the knife is to be carried across the anterior chamber. Fifthly, the point of the knife is to be directed upwards, and the handle downwards, so as to divide the cornea on its nasal side. Sixthly, in withdrawing the instrument, the upper and temporal portion of this incision is to be finished.

The operation of extraction is universally confessed to be a difficult one, the section of the cornea to be the finest manipulation in the practice of surgery. That the difficulties are to be lessened by the see-saw movements of Mr. Scott's knife, is, we think, extremely improbable. How much more simple, and more likely to answer the purpose intended, viz., a clean cut of the cornea, is the course of Beer's knife, which, from the first puncture to the end of the section, is in the same unvaried direction!

In several parts of his pamphlet Mr. Scott deprecates the employment of pressure. But is it not evident, that in the method described by himself, there must be, at different stages, very considerable pressure exercised? When, for instance, in the second step, he sweeps the handle of the knife up from its inclination towards the patient's face, the temporal side of the cornea must serve as a pivot on which the turn is to be made, and be considerably pressed upon. So much so, that the eyeball, unsupported, as Mr. Scott directs it to be, on the nasal side, must be exceedingly liable to be pushed into the internal canthus. In carrying the knife across the anterior chamber in the fourth step, Mr. Scott, contrary to the dread he formerly expressed (p. 34) of pressing the back of the knife against the undivided part of the cornea, at the two extremities of the incision, tells us, that "great care must be taken to press firmly downwards with the back of the instrument," (p. 43,) and we cannot see that the pressure of his curved knife is likely to be any less detrimental than that of a straight one. If it is the pressure of the knife, in the way mentioned, which excites the violent spasm of the muscles which Mr. Scott describes, the spasm will occur just as readily with the one instrument as the other. The fifth step is altogether one of pressure, and that in a most unfavorable direction, for the nasal portion of the incision is to be accomplished, not by the easy gliding on of the edge of the knife, but by raising its point, lowering its handle, and thus wrenching through the cornea. In every case, but especially in old subjects, in whom the cornea is so often hard and tough, giving rise to a grating sound when it is cut, this part of Mr. Scott's operation must be attended with dragging of the eye, and often fail, we should apprehend, in effecting the division of the cornea, which is intended.

The due retention of the aqueous humour in the eye, so as to avoid the entanglement of the edge of the knife by the iris, depends, as is well known, on the proper form of the knife, which, like a wedge, should accurately increase in breadth and thickness all the way from the point to the handle, and on the steadiness with which it is passed from one side of the cornea to the other, and onwards till it cuts itself out. The appropriate form of instrument, Mr. Scott says, (p. 36,) he retains enough to prevent the escape of the aqueous humour in its transit across the anterior chamber; but in the fifth step, when he raises the point of the knife, does he not leave a hiatus between the nasal extremity of the incision and the back of the instrument, by which the aqueous humour is almost sure to spring out? As for steadiness, he seems to set it at defiance, and recommends the section to be made, as we have seen, by an actual see-saw, or series of alternate motions of the instrument up and down. Beer's knife is steadied by its back resting at the extremities of the incision on the undivided portion of the cornea. This gives a precision to the motion of its edge, which we consider as of incalculable value. Not so Mr. Scott, who tells us, again and again, that his knife is "an instrument that accomplishes the division of the membrane independently of any such pressure." (p. 43.) The pressure, then, must be on its cutting edge alone, a mode of employing a cataract-knife which we consider as far from being manageable.

The last step of Mr. Scott's incision is accomplished by drawing the knife against the inside of the cornea, and out of the eye; and upon this our author founds the following strange comparison:

"Those who have ever performed the operation of lithotomy with the gorget, and afterwards with the small-beaked knife first used by the late Mr. Blizard, and have contrasted the force necessary to make the section of the prostate gland from without inwards by means of the former instrument, with the facility with which, the latter being introduced into the bladder, the section can be made from within outwards, will readily understand the advantages that attend the mode of operating I now propose, as well as the reasoning that has led to its adoption." (p. 36.)

At this rate, the best mode of cutting the cornea would be to transfix it with a narrow knife, and then, by drawing this out, make the section. The inevitable loss of the aqueous humour, and the violent dragging of the eye, so likely to cause bursting of the vitreous humour, are sufficient objections to any such mode of operating, and must attach themselves, in some degree, to Mr. Scott's operation, of which, although the greater part is performed on the same plan as that with the straight knife, namely, by cutting from without inwards, the termination is by an opposite movement, namely, from within outwards.

The section of the cornea, as every one knows, is divided into the puncturation, the counter-puncturation, and the completion.

As general principles, we can have no hesitation in laying it down, 1st, That the quantity of resistance to the penetration and progress of a knife through the cornea, will increase and diminish, *cæteris paribus*, with the augmented or diminished angle of inclination of its edges; and 2d, That there is no curved figure, convex or concave, assignable to the cutting edge of the knife, by which the friction can be reduced below that of a straight line.

Although the penetration of the cornea will be effected with least resistance by a straight knife, the edges having the smallest possible inclination to one another, it is evident that a very narrow straight instrument is not at all calculated for completing the section; and that for the following reasons:

1st. It cannot complete the section, or cut itself out, as the phrase is, without letting out the aqueous humour.

2d. It is much more difficult to continue the section in one plane with a narrow than with a broad instrument.

3d. A narrow instrument must at last be used saw-wise.

4th. A narrow instrument, as the incision proceeds, comes to cut the cornea, not edgewise, but sidewise. Let the space between the two circles, in the six following diagrams, represent the thickness of the cornea.

fig. 2.

fig. 3.

fig. 4.

fig. 5.

fig. 6.

fig. 7.

At *a* and *á* (fig. 2,) the instrument is cutting through a thin edge, but at *b* (fig. 3,) through a broad surface; and the proportional difference will be greater the thinner the membrane. In fig. 2, the knife is dividing the laminæ of the cornea nearly perpendicularly; in fig. 3, it is acting nearly parallel to the laminæ. One of the great advantages of a broad knife is, that it continues to cut the cornea in some degree edgewise, or perpendicularly to its laminæ, till the incision is completed.

It follows, that a narrow knife is best for penetrating, a broad one best for cutting itself out, or completing the section. Experience alone can decide the form which will combine the two qualities most perfectly. Every one who has extracted knows, that the great resistance is at the completion of the incision, when the motion of the knife is nearly parallel to the laminæ. We believe that the angle of inclination to be given to the edges of the knife, so that it may accomplish the incision with least resistance, and least extent of motion, is about 15°.

As for a narrow curved instrument, cutting by its convex edge, it seems liable to many objections, which do not apply to a straight instrument.

1st. Puncturation offers a great resistance, except it be in the line of the cutting edge of the instrument. In order to puncture the cornea easily with a curved instrument, the motion must be in the direction of the curve, the laminæ must actually be cut in a curve, which causes a greater resistance. Besides, it is far more difficult to follow one precise curve than a straight line. Fig. 4 shows puncturation with a curved knife. To be performed with ease, the instrument must follow the course of the dotted line.

2d. With a curved knife, the difficulty of the counterpuncturation is much increased. The direction of the knife must be shifted, in order to come into contact with the proper point of the cornea, and when that point has been hit, as in fig. 5, the direction of the motion must again be changed, as is shown by the dotted line; otherwise, the point of the knife, attempted to be carried on in a straight line, will meet with an enormous resistance. In short, the resistance, both in puncturation and counter-puncturation, is necessarily greater with a curved than with a straight instrument, and is increased beyond measure, if the force deviates in direction from the line of the curve.

3d. In carrying the knife across the anterior chamber, it is of great importance to keep its edges always in the same plane, as any bias which they are allowed to take, different from the direction which they had when the counter-puncturation was effected, is apt to allow an escape of the aqueous humour. We are ready to grant, that it is easier to keep a narrow curved instrument in the same plane than a narrow straight one.

4th. The great disadvantage of a curved knife is, that, in completing the section, it comes to act sidewise against the cornea, or, in other words, parallel to the laminæ to be divided, much sooner than the ordinary triangular knife. The retreating of the curved knife from the perpendicular direction, as at *a*, fig. 6, and *á*, fig. 7, is a most serious defect; and the statement of Mr. Scott, that his curved knife is better fitted than Beer's straight knife, to "be opposed only to the margin of the section on either side, and not to any extensive portion of its internal surface," (p. 36,) is manifestly opposed to the plainest mathematical principles, as will be seen by comparing fig. 7 with fig. 3. The great point in a cataract knife is to have the cutting edge as nearly perpendicular to the course of the incision as possible. In such an instrument as Mr. Scott's, the whole contrivance is adapted to make the cutting edge as nearly as possible parallel to the course of the incision, and exactly the reverse of what the inventor says he intended.

Here we must drop the subject of cataract knives, as we doubt not that many of our readers will feel but little interest in matters so minute and technical.

That Mr. Scott can operate with his curved knife, and operate well too, especially when his cutler happens to make it sharp, which, from its shape, can rarely happen, we have no manner of doubt; but, both on theoretical principles, and from practical knowledge, we should advise those who are commencing practise on the eye, to avoid so unphilosophical and dangerous an implement, and keep to the triangular knife of the justly-celebrated Professor Beer, as an instrument better calculated than any other hitherto invented, to enable them to finish the section of the cornea, *tuto, cito, ac jucunde*.

Mr. Scott disapproves of bleeding the patient who is about to submit to extraction, with the view of preventing inflammation. Neither does he bleed after the operation, unless urgent inflammatory symptoms occur, and the patient is robust and able to bear depletion. He advises every precaution to be followed which can tend to bring the patient beforehand into the most favorable condition for the operation, (p. 45,) but omits to say in what such precautions consist.

The most frequent accident after the operation, according to Mr. Scott, is protrusion of the iris through the wound of the cornea. (p. 46.) He ascribes this to spasms of the muscles, excited by any slight blow on the eye, and recommends the protruding membrane to be touched with nitrate of silver.

Of inflammatory affections following extraction, Mr. Scott distinguishes the following varieties:

1. That which rises from under violence in operating, characterized by increased vascularity of the sclerotica, as well as of the conjunctiva, haziness of the cornea, and dulness of the iris. He says it is attended also by an effusion of *lymph* under the conjunctiva, but we see no reason to believe that the effusion is different in this case from what it is in any other instance of chemosis, that is to say, *serous.* Leeches are chiefly recommended, so as to effect a gradual abatement of the symptoms; warm fomentations; and belladonna. We should deem Mr. Scott's fear, that the too early application of belladonna keeps up the inflammation of the iris, and separates the adhesion too rapidly, perfectly groundless. No mention is made of mercury for this variety.

2. A tedious inflammation, difficult to control, sometimes follows the operation, when the eye has been previously the seat of some inflammatory affection. Moderate depletion is advised, a nutritious diet, and a mild use of mercury. Mr. Scott makes some remarks on the wonderful powers of mercury, admitting the facts, first announced to the profession by Beer,* and now so generally known and acted on, that when there is too much inflammation, this medicine so controls the action of the vessels, as to *prevent the effusion* of lymph into the eye, and that if lymph is already effused, it *promotes its absorption.* He also notices the non-interference of mercury with the healing process, in cases of wounds and ulcers, a doctrine much insisted on by the late Professor Hamilton of Edinburgh.† If this medicine can both stimulate the absorbents to take up lymph, when it is superabundant, and, lulling them asleep when they are too active, as in ulceration, can repair the destruction they have effected, by promoting a healing process, besides controlling the morbid action of the blood-vessels, if used in the earlier stages of inflammation, we shall almost be persuaded into the belief of an *animus medicatrix mercurii.* The truth is, our theory of morbid actions is too imperfect, to enable us to comprehend the effects of mercury; but the fact, that it acts beneficially, in states of disease which seem totally opposed to one another, does not admit of denial.

3. A morbid irritability of system, consequent on want of power in the constitution, produces a form of inflammation, which Mr. Scott describes as coming on two or three days after the operation, especially in old subjects. It is attended with intense pain, of a throbbing aching character, extending deep into the orbit, and affecting the temple, succeeded by great tumefaction of the eyelids, which assume a livid hue, and yellowish-red chemosis of the conjunctiva. The pulse is quick, weak, and small, and the surface of the body pale and cold. The cornea is hazy, and the edges of the wound tumid and of a dirty yellowish colour. Warm fomentations constitute the chief local application. Opium, with ether or ammonia, is to be given internally, to allay the irritability and restlessness. The powers of the system are to be sustained by nutritious diet, cordials, and stimulants.

4. When acute phlegmonous inflammation of the globe occurs, which it does very rarely, it commences within a few hours after the operation, with pain of an acute and throbbing character; the eye is exquisitely sensitive to touch; the pain extends deep into the orbit, and to the side

* Beer's Bibliotheca Ophthalmica, vol. i, p. 55, and vol. ii, p. 85.—Vindobonæ, 1799. Also his Lehre von den Augenkrankheiten, vol. i, p. 449.—Wien, 1813.

† Hamilton's Observations on the Use and Abuse of Mercurial Medicines, p. 219.—Edinburgh, 1819.

of the head, and gradually increases in severity, without intermission or abatement. The eyelids assume a bright red, not a livid hue ; they become somewhat swollen, and the surface of the globe prominent from chemosis, but not so rapidly, nor to the same extent as in the third variety of inflammation. If the disease be not checked, enormous tumefaction of the eye supervenes, followed by sloughing of the cornea and suppuration of the ball. A hard, full, throbbing pulse, a hot and dry skin, and the other symptoms of fever are present. By active depletion, an attempt must be made to cut short the inflammation, else the eye will be lost. Free venesection and numerous leeches to the eyelids are chiefly to be depended on. Calomel and saline aperients are to be administered. Abstinence and rest in bed are to be enjoined.

5. In gouty or rheumatic subjects inflammation is apt to continue after the section is healed, and produce closure of the pupil. Mr. Scott ascribes this to too great a reduction of the patient by low diet. He advises cupping on the temple, a blister to the nape, drastic purges, bark, and belladonna. A nutritious but not stimulating diet is to be given, and the patient properly defended from vicissitudes of temperature. The eye is to be kept closed, and all lotions and fomentations avoided. The digestive organs are generally wrong, and may require an alterative course of mercury ; but the system is not to be affected, as this increases irritability and aggravates the inflammation.

We give this abstract of Mr. Scott's account of the different varieties of inflammation after extraction, as we deem such distinctions of high practical importance, and deserving of more attention than is generally bestowed on them.

The operation of displacement recommended by Mr. Scott, and called by him depression, but which, he says, is a combination of reclination with depression, (p. 56,) is Willburg's reclination performed with Scarpa's needle.

As the operation is apt to excite inflammation of the internal tunics of the eye, " the patient should be in a condition the least prone to inflammation ; consequently," says Mr. Scott, " it is necessary to adopt beforehand all the precautions that were mentioned in speaking of the operation of extraction." But, unfortunately, no particular precautions are mentioned, as far as we have been able to discover.

Dilating the pupil before operating, Mr. Scott regards as of no importance. " I have never found any difficulty," says he, " in operating in the contracted state of the pupil, in which state you lessen the risk of the lens becoming dislocated and wedged in the aperture." (p. 54.)

Mr. Scott directs the needle to be introduced " about a line behind the margin of the iris," at which distance he will barely avoid the ciliary processes, " and a little lower than the transverse axis of the globe, to avoid," he says, " the ciliary artery," but where it is likely to meet one of the primary branches of that artery, which divides into two about three lines from the cornea.

After describing the common mode of operating, in which, neglecting to open the posterior capsule, but freely lacerating the anterior, the operator turns the lens over into the lower part of the vitreous humour, Mr. Scott gives an account of another method of displacing the lens, which has originated, it seems, with Mr. Egerton of Calcutta, and which consists in pushing a straight needle into the edge of the lens, pressing downwards and backwards, so as to burst the posterior capsule, and lodge the lens behind the ciliary processes, with its posterior surface up and its anterior surface down, and then freeing the instrument from the lens by rotating it on its axis. This is a mode of operating which we think inferior, in point both of ingenuity and safety, to that of Sautconnea, the native oculist of Calcutta, of whom Mr. Breton has given so interesting an account.* Mr. Scott deems Mr. Egerton's operation objectionable unless something more is done than is mentioned above, because, says he, "the anterior capsule will probably be left entire, and if it be not previously opaque it will necessarily become so after the operation." He therefore recommends the anterior capsule to be immediately lacerated through the cornea, judging this preferable to any attempt to divide it with the needle employed through the sclerotica for the displacement, as the doing so might endanger the reascension of the lens. (p. 57.)

We must confess we are somewhat astonished to find Mr. Scott asserting, that if the anterior capsule be left entire it will necessarily become opaque, when it is well known that some of the most experienced and successful operators have made it an invariable rule to leave the anterior capsule entire.† We have no hesitation in stating, that neither in the entire nor in the lacerated state does the anterior capsule become opaque, unless as a consequence of inflammation supervening to the operation. In a little work by Dr. D. W. Soemmerring, entitled, 'Observations on the Organic Changes which occur in the Eye after Operation for Cataract,' published in German in 1828, and abounding with the most valuable information, the interior of an eye is represented in which the lens had been reclined eight years and a half before the death of the patient. The anterior capsule was found in the state of two transparent semilunar flaps.

If either of Mr. Scott's two plans of displacing the cataract be followed, and the aqueous and vitreous cavities of the eye converted into one, by lacerating the anterior capsule, the result is likely to be, *in the first place*, much more severe inflammation of the interior textures—especially of the choroid and iris—than if the anterior capsule had been left entire; and, *secondly*, a reascension, sooner or later, of the lens, which now lies at the bottom of the eye, surrounded not so much by the vitreous humour as by the aqueous, which, being probably more rapidly secreted than the vitreous, speedily infiltrates the hyaloid cells, and fills the cavity left by the revolution of the lens into its new situation.

Mr. Scott speaks of inflammation of the internal tunics of the globe, and of reascension of the lens as frequent results of the operation of displacement. Nor is this at all surprising, after the cavities of the eye are jumbled into one, by breaking down the partition which nature has placed between them.

* On the Native Mode of Couching. By Peter Breton.—Asiatic Lithographic Press, 1826.

† Acus oculo ad deponendam suffusionem immersa, et per pupillam conspicua, nusquam inter iridem et tunicam crystallinam quidquid vulgo existimatur, sed pone hanc ipsam consistit, ita ut tunica prædicta integra, sana, et sine ulla ab operatione plaga facile servari queat, modo caveatur, ne cuspis acus rursus iridem, pupillam, aut cameram oculi anteriorem, ut incaute fieri solet, agatur. Ferrein, in Haller's Disputationes Chirurgicæ, tom. v, p. 569.—Lausannæ, 1756. See also Taylor's Treatise on the Diseases of the Crystalline Humour, p. 33.—London, 1736.

Mr. Scott's appellation for division of the cataract is, *The operation by solution*, which might lead many, ignorant of the subject, to suppose that the operation was effected by solution, rather than the solution brought about by operation.

One of the interesting questions which still remain to be decided in the surgery of the eye, is the proportion of cataracts curable by solution. How frequently do we hear oculists exclaim, after an extraction is finished, and they take up the lens on the point of the capsule-needle, "Well! this is much softer than I had suspected. Had I thought it so soft, I should have divided it!"

On the one side we may, perhaps without injustice, place our present author. He tells us, that if extraction cannot be performed, "the cataract being hard, depression must be had recourse to." (p. 53.) Of the proportionate number of hard and soft cataracts he says nothing, but it is plain he is unfavorable to the operation of division, except when the lens is so soft as readily to admit of its texture being opened up by the needle.

"The operation by solution," he says, "should be confined to soft cataracts. If you attempt in this way to remove a hard cataract, several years will often elapse before its entire removal can be accomplished, and the operation will require to be so frequently repeated, that there will be great risk of its producing inflammation. When the volume of the lens has been thus diminished, there will be greater danger of the hard nucleus becoming dislocated, so as to press upon the pupillary margin of the iris and induce very serious inflammation, which sometimes will not subside until the iris is relieved from pressure by extracting the lens." (p. 61.)

On the other side, we may place Dr. Jacob, of Dublin, who, speaking of division, says:

"It is urged as an objection to this operation, that it is applicable to cases of soft cataract only. Whatever meaning may be attached to the term soft cataract, my experience leads to the conclusion, that the operation, properly modified, is applicable to the great majority of cases, perhaps to nine in ten. It is said that it often requires to be repeated; but this is a minor evil, to which we submit, in preference to incurring the risk of either of the other operations. Extraction, if unsuccessful, cannot be repeated, and a repetition of depression is not very desirable. It has been said, without the least foundation in truth, that vision is not so perfect after this as after other operations; the reverse is, I believe, generally speaking, the fact. That more time elapses between the performance of the operation and the recovery of sight than in the other operations must be admitted, but this, which may be a very valid objection on the part of metropolitan oculists, many of whose patients come from a distance, cannot be considered of great importance elsewhere, the disadvantage of delay being counterbalanced by the greater security afforded by the mildness of the operation." *

As in many similar controversies, so here, the truth lies between. Many eyes are lost by extraction, which might have been saved by division; and many destroyed by division, for which extraction alone would have been the proper operation.

Mr. Scott prefers division through the cornea rather than through the sclerotica. He says it is immaterial whether the pupil be dilated by belladonna or not. Surely the iris is less likely to be injured, if the pupil is dilated; and perhaps it is as well, in such an operation, as in most others, to see what one is about, which the dilatation of the pupil enables us to do. Mr. Scott prefers a straight needle, which is not so well adapted

* Dublin Hospital Reports, vol. iv. p. 216.—London, 1827.

for comminuting the capsule as a curved one. A needle of the general construction of Dr. Jacob's, but not quite so much curved, and not chisel-shaped, but pointed, we consider as the best.

Mr. Scott follows Conradi's plan, of one incision only through the capsule; but after a single incision, absorption goes on extremely slowly, and, if the slightest inflammation occurs, the wound of the capsule is apt to close.

Division through the sclerotica, as described by Mr. Scott, is the exploded operation of Sir William Adams, with all its gross absurdities; such as, entering the needle with its flat side parallel to the iris, cutting the lens into two halves, &c. Either Mr. Scott is totally unacquainted with any better mode of performing the posterior operation of division than this, or he chooses to describe an operation which none but a madman would attempt at the present day, as a contrast to Conradi's anterior operation, which he himself has adopted.

The pamphlet terminates with some remarks on capsular cataract, and on the use of cataract-glasses. What is said on the latter head is of the most common-place description. Mr. Scott says, "there is some variety in the focus of different glasses of the same number," but this cannot be; for the numbers of convex glasses are not arbitrary, as is the case with those of concave glasses, but express, in inches and parts of an inch, the focal length. A few glass-vendors may, indeed, mark a glass as of four and a half inches when it is of five, but a lie of this sort is detected in a moment.

## Figures and Tables

**Fig. 1. f1:**
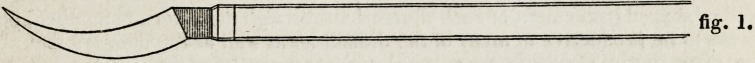


**Fig. 2. f2:**
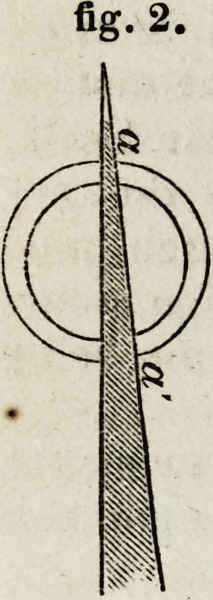


**Fig. 3. f3:**
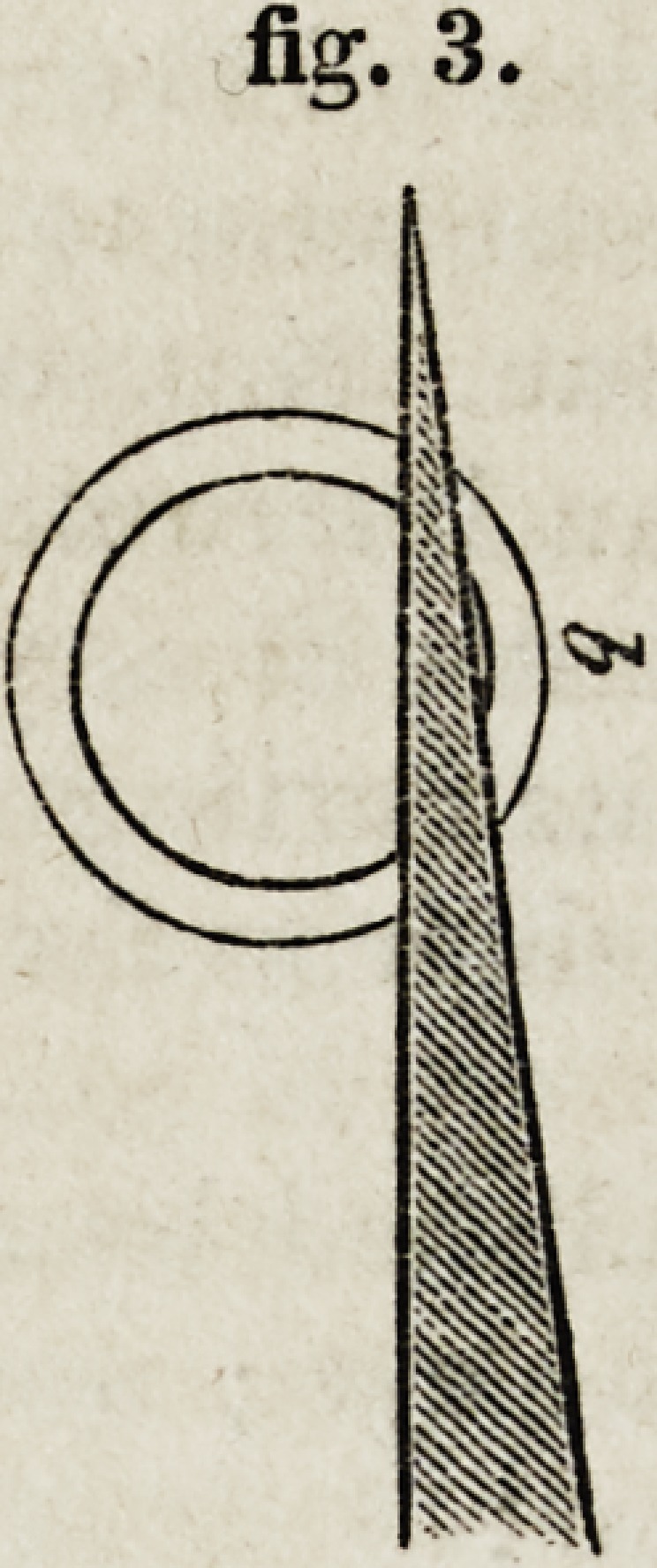


**Fig. 4. f4:**
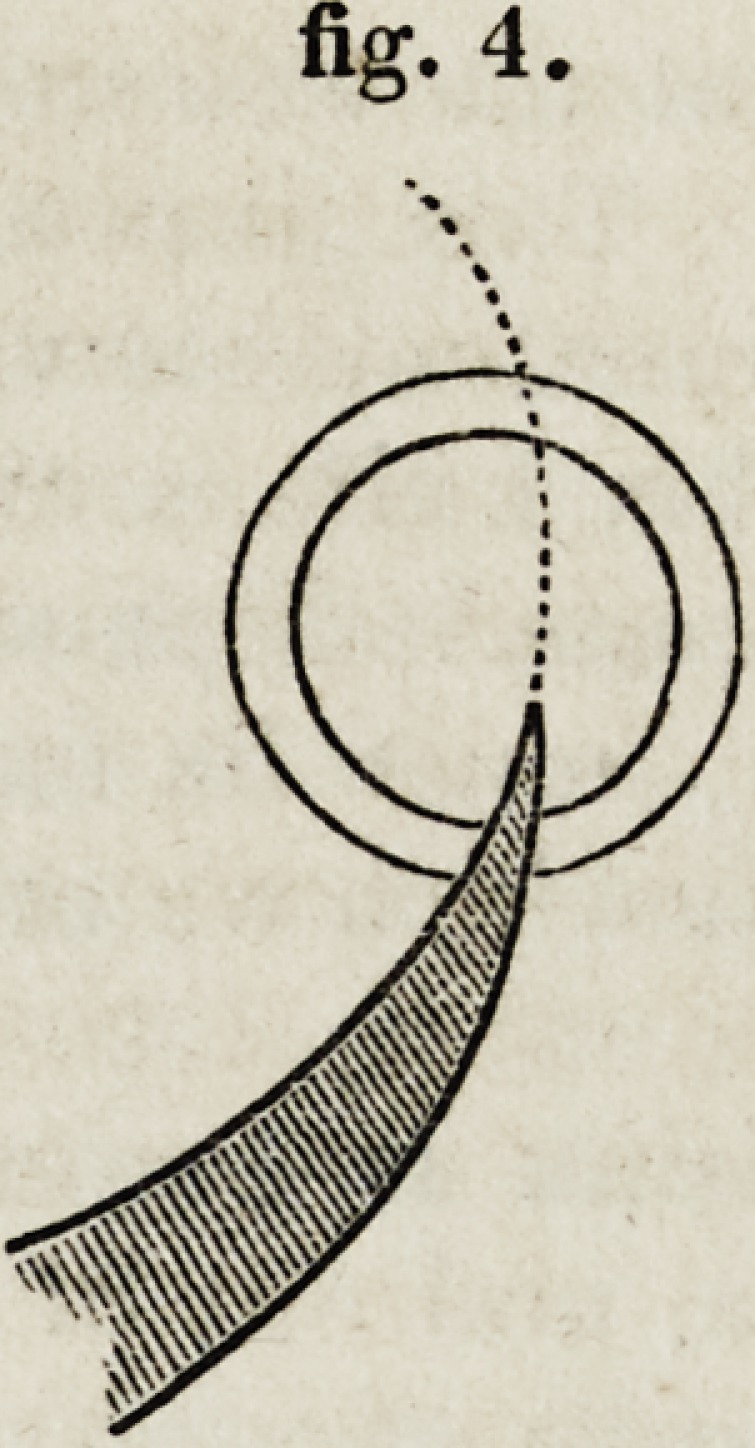


**Fig. 5. f5:**
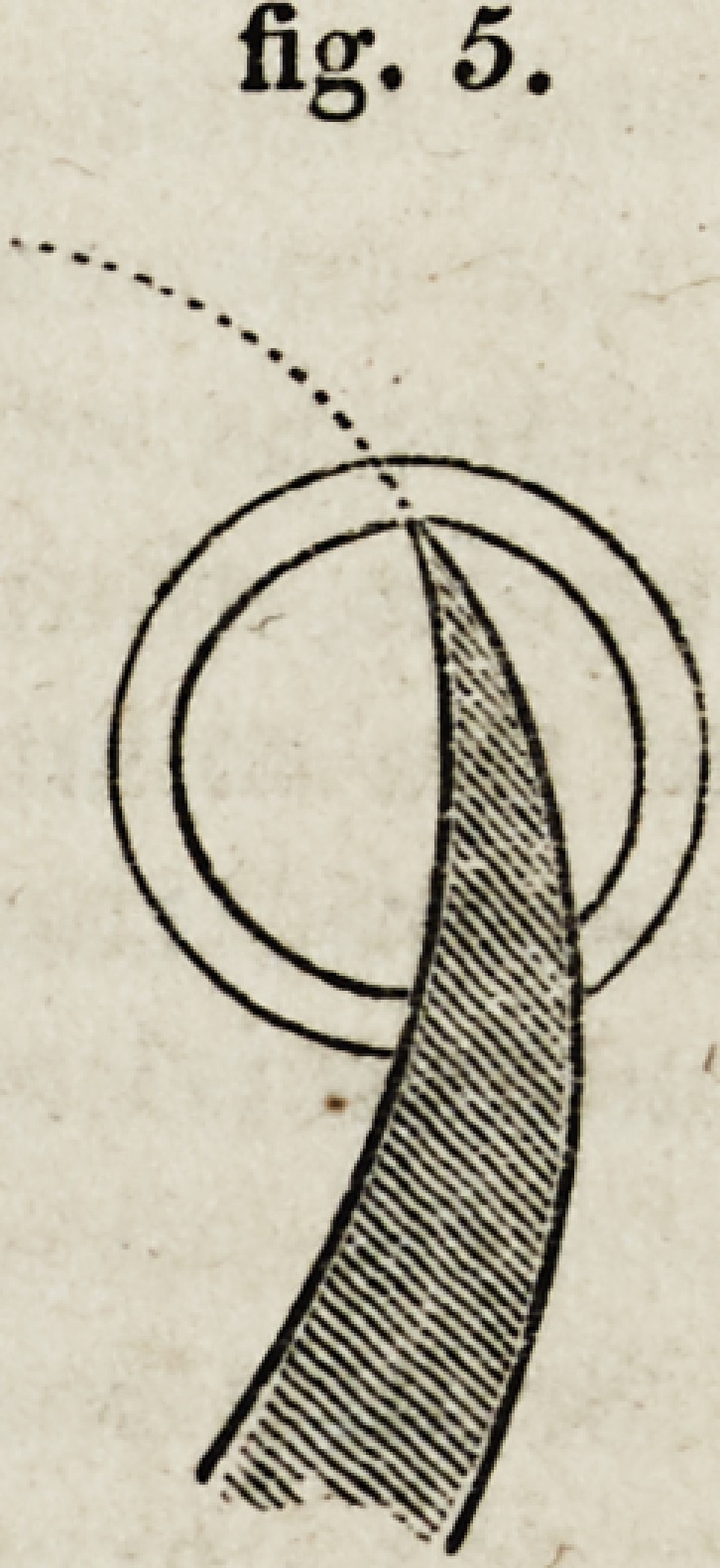


**Fig. 6. f6:**
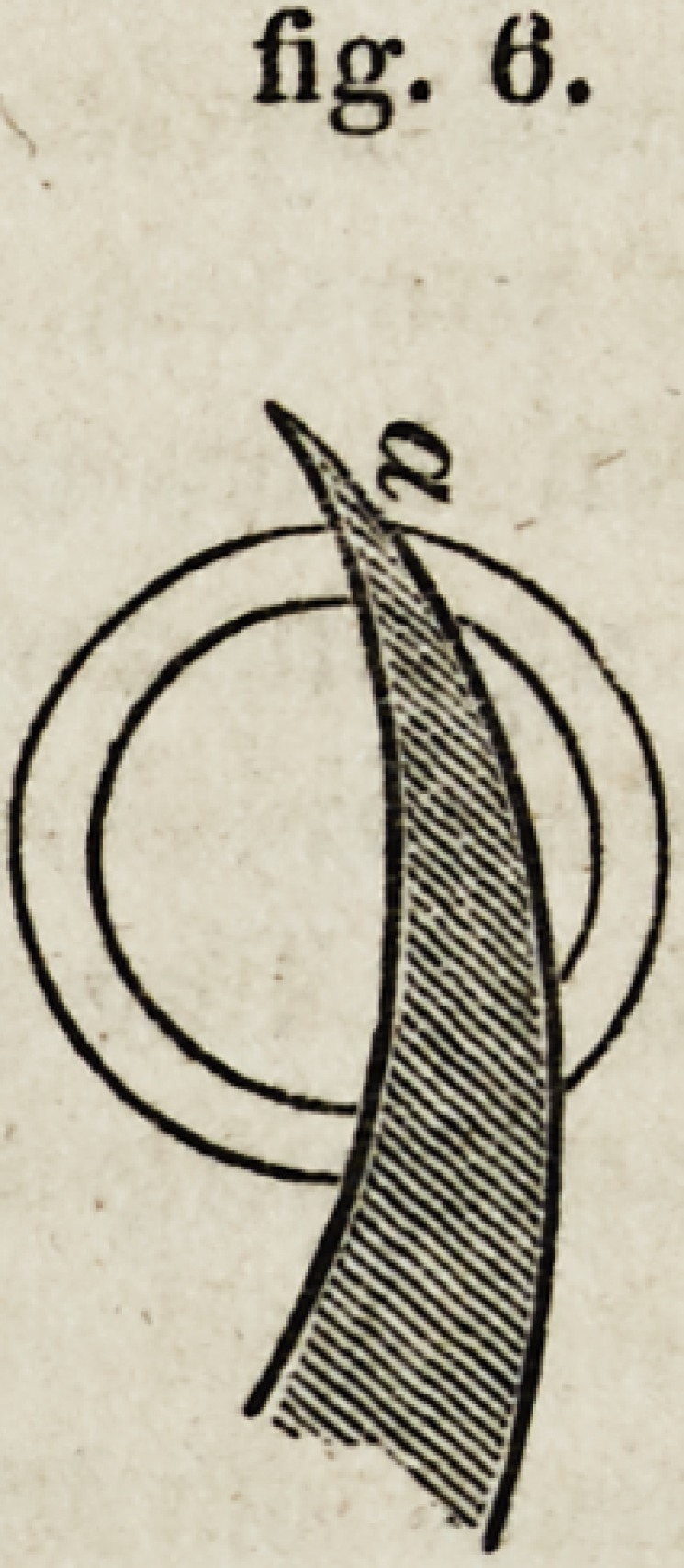


**Fig. 7. f7:**